# Evidence of recent natural selection at Alzheimer Disease risk loci and implications for precision medicine

**DOI:** 10.1002/alz70855_107509

**Published:** 2025-12-29

**Authors:** Timothy H Ciesielski, Neetesh Pandey, Farid Rajabli, Himiede Sesay, Razaq O Durodoye, Rufus O. Akinyemi, Margaret Pericak‐Vance, Maryenela Z. Illanes‐Manrique, Mario Cornejo‐Olivas, Nilton Custodio, Rafael Samper‐Ternent, Rebeca Wong, Jonathan L Haines, Giuseppe Tosto, Scott M Williams

**Affiliations:** ^1^ Case Western Reserve University School of Medicine, Cleveland, OH, USA; ^2^ Columbia University, New York, NY, USA; ^3^ John P. Hussman Institute for Human Genomics, University of Miami Miller School of Medicine, Miami, FL, USA; ^4^ Case Western Reserve Uinveristy School of Medicine, Cleveland, OH, USA; ^5^ College of Medicine, University of Ibadan, Ibadan, Oyo State, Nigeria; ^6^ Institute of Advanced Medical Research and Training, College of Medicine, University of Ibadan, Ibadan, Oyo State, Nigeria; ^7^ Neurogenetics Working Group, Universidad Científica del Sur, Lima, Peru; ^8^ Neurogenetics Working Group, Universidad Cientifica del Sur, Lima, Peru; ^9^ Unidad de Investigación, Instituto Peruano de Neurociencias, Lima, Lima, Peru; ^10^ University of Texas Medical Branch, Galveston, TX, USA; ^11^ University of Texas, San Antonio, TX, USA; ^12^ Case Western Reserve University School of Medicine, Department of Population & Quantitative Health Sciences, Cleveland Institute for Computational Biology, Cleveland, OH, USA; ^13^ Department of Population & Quantitative Health Sciences, School of Medicine, Case Western Reserve University, Cleveland, OH, USA; ^14^ Taub Institute for Research on Alzheimer's Disease and the Aging Brain, Vagelos College of Physicians and Surgeons, Columbia University, New York, NY, USA; ^15^ Department of Neurology, College of Physicians and Surgeons, Columbia University, and the New York Presbyterian Hospital, New York, NY, USA

## Abstract

**Background:**

Many of the over 70 single nucleotide polymorphisms (SNPs) that associate with late‐onset Alzheimer (LOAD) are common. LOAD risk alleles may achieve high frequency by providing beneficial effects earlier in life for other, as yet undefined, phenotypes. These beneficial effects and the natural selection they drive may differ across populations, and if so, identifying the LOAD risk variants under selection in distinct ancestry groups could illuminate how LOAD etiology differs between these groups. Therefore, we tested for recent selection in a comprehensive list of LOAD risk loci across 26 populations worldwide. Differences here would indicate that comorbidity patterns, intervention efficacies, and off‐target effects could differ by ancestry group.

**Method:**

We obtained whole‐genome sequence data from 2,504 participants in the 1000 Genomes Project and used Selscan to obtain genome‐wide nSL scores (a measure of recent evolutionary selection) for each of the 26 populations. We then extracted the top 1% of |nSL| and identified sites within or near LOAD associated genes using annotated lists. Then we obtained nSL scores from five racial/ethnic(R/E) groups among 15,264 controls from the Alzheimer Disease Sequencing Project(ADSP). Loci were considered validated if they had a site in the top1% of |nSL| scores in a corresponding R/E group.

**Results:**

Of the 75 LOAD associated loci, 52 had at least one variant in the top1% of |nSL| in two populations from the 1000 Genomes data. Among ADSP controls, 26 of these 52 loci were validated in a corresponding R/E group. *ICA1* showed evidence of selection in 22 of 26 populations and was validated in all ADSP racial‐ethnic groups except African Americans. Validated loci differed by superpopulation (African/European/South‐Asian/Amerindian), and pathway analyses implicated distinct biological processes. For example, selection on microtubule function was more prominent in Africans.

**Conclusions:**

At least 26 LOAD risk loci show evidence of recent evolutionary selection. Pathway analyses indicate that evolution may be acting on distinct biological functions in each superpopulation. Selection on microtubule function in Africans may align with observed differences in Tau physiology in African Americans. Thus, LOAD etiology and comorbidity/pre‐morbidity patterns may differ by superpopulation. Research and interventions may need to be tailored.

